# Doxorubicin Exposure and Breast Cancer Risk in Survivors of Adolescent and Adult Hodgkin Lymphoma

**DOI:** 10.1200/JCO.23.01386

**Published:** 2024-02-15

**Authors:** Suzanne I.M. Neppelenbroek, Yvonne M. Geurts, Berthe M.P. Aleman, Pieternella J. Lugtenburg, Saskia E. Rademakers, Roel J. de Weijer, Maaike G.A. Schippers, Bastiaan D.P. Ta, Wouter J. Plattel, Josée M. Zijlstra, Richard W.M. van der Maazen, Marten R. Nijziel, Francisca Ong, Erik C. Schimmel, Eduardus F.M. Posthuma, Marie José Kersten, Lara H. Böhmer, Karin Muller, Harry R. Koene, Liane C.J. te Boome, Yavuz M. Bilgin, Eva de Jongh, Cécile P.M. Janus, Flora E. van Leeuwen, Michael Schaapveld

**Affiliations:** ^1^ Department of Epidemiology, Netherlands Cancer Institute, Amsterdam, the Netherlands; ^2^ Department of Radiation Oncology, Netherlands Cancer Institute, Amsterdam, the Netherlands; ^3^ Department of Hematology, Erasmus MC Cancer Institute, University Medical Center Rotterdam, Rotterdam, the Netherlands; ^4^ Department of Radiation Oncology, Leiden University Medical Center, Leiden, the Netherlands; ^5^ Department of Hematology, University Medical Center Utrecht, Utrecht, the Netherlands; ^6^ Department of Radiation Oncology, Instituut Verbeeten, Tilburg, the Netherlands; ^7^ Department of Radiation Oncology (Maastro), GROW School for Oncology, Maastricht University Medical Centre+, Maastricht, the Netherlands; ^8^ Department of Hematology, University Medical Center Groningen, University of Groningen, Groningen, the Netherlands; ^9^ Department of Hematology, Amsterdam UMC, Location Vrije Universiteit, Cancer Center Amsterdam, Amsterdam, the Netherlands; ^10^ Department of Radiation Oncology, Radboud University Medical Center, Nijmegen, the Netherlands; ^11^ Catharina Cancer Institute, Department of Hemato-Oncology, Catharina Hospital, Eindhoven, the Netherlands; ^12^ Department of Radiotherapy, Medisch Spectrum Twente, Enschede, the Netherlands; ^13^ Department of Radiotherapy, Radiotherapiegroep, Arnhem, the Netherlands; ^14^ Department of Internal Medicine, Reinier de Graaf Hospital, Delft, the Netherlands; ^15^ Department of Hematology, Amsterdam University Medical Centers, Location University of Amsterdam, Cancer Center Amsterdam, Amsterdam, the Netherlands; ^16^ Department of Hematology, Haga Teaching Hospital, Den Haag, the Netherlands; ^17^ Department of Radiotherapy, Radiotherapiegroep, Deventer, the Netherlands; ^18^ Department of Hematology, St Antonius Hospital, Nieuwegein, the Netherlands; ^19^ Department of Hematology, Haaglanden Medical Center, Den Haag, the Netherlands; ^20^ Department of Internal Medicine, Admiraal De Ruyter Hospital, Goes, the Netherlands; ^21^ Department of Internal Medicine, Albert Schweitzer Hospital, Dordrecht, the Netherlands; ^22^ Department of Radiotherapy, Erasmus MC Cancer Institute, University Medical Center Rotterdam, Rotterdam, the Netherlands

## Abstract

**PURPOSE:**

Female Hodgkin lymphoma (HL) survivors treated with chest radiotherapy (RT) at a young age have a strongly increased risk of breast cancer (BC). Studies in childhood cancer survivors have shown that doxorubicin exposure may also increase BC risk. Although doxorubicin is the cornerstone of HL chemotherapy, the association between doxorubicin and BC risk has not been examined in HL survivors treated at adult ages.

**METHODS:**

We assessed BC risk in a cohort of 1,964 female 5-year HL survivors, treated at age 15-50 years in 20 Dutch hospitals between 1975 and 2008. We calculated standardized incidence ratios, absolute excess risks, and cumulative incidences. Doxorubicin exposure was analyzed using multivariable Cox regression analyses.

**RESULTS:**

After a median follow-up of 21.6 years (IQR, 15.8-27.1 years), 252 women had developed invasive BC or ductal carcinoma in situ. The 30-year cumulative incidence was 20.8% (95% CI, 18.2 to 23.4). Survivors treated with a cumulative doxorubicin dose of >200 mg/m^2^ had a 1.5-fold increased BC risk (95% CI, 1.08 to 2.1), compared with survivors not treated with doxorubicin. BC risk increased 1.18-fold (95% CI, 1.05 to 1.32) per additional 100 mg/m^2^ doxorubicin (*P*_trend_ = .004). The risk increase associated with doxorubicin (yes *v* no) was not modified by age at first treatment (hazard ratio [HR]_age <21 years_, 1.5 [95% CI, 0.9 to 2.6]; HR_age ≥21 years_, 1.3 [95% CI, 0.9 to 1.9) or chest RT (HR_without mantle/axillary field RT_, 1.9 [95% CI, 1.06 to 3.3]; HR_with mantle/axillary field RT_, 1.2 [95% CI, 0.8 to 1.8]).

**CONCLUSION:**

This study shows that treatment with doxorubicin is associated with increased BC risk in both adolescent and adult HL survivors. Our results have implications for BC surveillance guidelines for HL survivors and treatment strategies for patients with newly diagnosed HL.

## BACKGROUND

Female Hodgkin lymphoma (HL) survivors treated with chest radiotherapy (RT) at a young age have a strongly increased risk of subsequent breast cancer (BC).^1,2^ Previous studies have shown that BC risk is higher after treatment at a younger age, with higher radiation doses and/or larger radiation volumes. Among women exposed to chest RT at age younger than 21 years, reported risks are comparable with those observed in *BRCA* mutations carriers.^2-7^ Several studies in HL survivors or childhood cancer survivors (CCS) reported a lower risk of radiation-associated BC among women who also received gonadotoxic treatments with high doses of alkylating agents (ie, procarbazine), or pelvic RT, compared with those who did not receive such treatments.^8-10^ This reduction of radiation-associated BC risk after gonadotoxic treatment has been attributed to a protective effect of early menopause. To decrease HL treatment-related gonadotoxicity, doses of alkylating chemotherapeutic agents and the use of pelvic RT have been reduced since the 1980s, while anthracyclines, such as doxorubicin, have become the cornerstone of chemotherapy for HL.^11-13^ Simultaneously, the volumes and doses used for chest RT have decreased.^14-16^ However, a previous study from our group among 5-year HL survivors treated between 1965 and 2000 did not demonstrate a lower BC risk in more recently treated patients.^17^

**CONTEXT**

**Key Objective**
To evaluate whether doxorubicin exposure is associated with breast cancer (BC) risk in female 5-year Hodgkin lymphoma (HL) survivors treated at age 15-50 years, between 1975 and 2008.
**Knowledge Generated**
Almost one in five survivors developed BC after 30 years of follow-up. Exposure to a cumulative doxorubicin dose of >200 mg/m^2^ was associated with a 1.5-fold increased BC risk, independent of age at HL treatment, receipt of chest radiotherapy (RT), or gonadotoxic therapy. Despite lower doses and smaller volumes of RT in contemporary treatment regimens, BC risk has not decreased in survivors treated between 1998 and 2008 compared with survivors treated between 1975 and 1986 (20-year cumulative BC incidence 7.8% *v* 8.2%, respectively).
**Relevance *(J.W. Friedberg)***
These results emphasize the importance of very long follow-up for survivors of HL, and, if validated in other datasets, provide another factor to consider when evaluating risks of therapy. Ongoing trials incorporating checkpoint blockade and decreasing chemotherapy exposure have particular relevance given these findings.*
  *Relevance section written by *JCO* Editor-in-Chief Jonathan W. Friedberg, MD.

Several studies in CCS populations reported increased BC risk after exposure to doxorubicin with and without chest RT,^18-22^ with data suggestive of dose-dependent increase in BC risk.

To the best of our knowledge, the association between doxorubicin exposure and BC risk has not yet been examined in HL survivors treated at adult ages. If doxorubicin exposure at adult ages were associated with BC risk, this could have implications for follow-up care of HL survivors, as well as for treatment strategies for newly diagnosed patients. Therefore, we assessed the effect of doxorubicin exposure on BC risk using a large cohort of female 5-year HL survivors, treated between 1975 and 2008, with a broad age range at the start of treatment.

## METHODS

### Study Population and Data Collection

This multicenter study cohort comprised 1,964 female 5-year survivors who had HL as their first malignancy and were treated in 20 Dutch centers (eight university medical centers, nine general hospitals, and three RT centers) in the Netherlands at age 15-50 years, between 1975 (when doxorubicin was introduced in HL treatment) and 2008.

Detailed information regarding HL diagnosis, and primary and relapse treatment(s) (radiation fields, chemotherapy regimens, and number of cycles) was collected from medical records as described previously.^23-25^ Cumulative doxorubicin and procarbazine doses were estimated on the basis of the number of cycles of prescribed chemotherapy regimens using standard dosages for each regimen.

For follow-up years until 1989, information on subsequent primary malignancies was collected directly from medical records and from questionnaires sent to general practitioners (questionnaire response until 1989: 96%).^26^ For follow-up from 1989 onward, information on subsequent BC was obtained through record linkage with the Netherlands Cancer Registry (NCR), which has nationwide coverage since that year.^27,28^

Vital status and date of death were obtained through linkage with the NCR and through record linkage with the Central Bureau for Genealogy. For survivors treated from 1989 onward, the most recent linkage with NCR was complete until January 1, 2022. For survivors treated between 1975 and 1988, the last NCR linkage was performed in 2012, as current European privacy regulations complicate new linkages for these survivors.

### Statistical Analysis

As only 5-year survivors were included in this study, time at risk started 5 years after start of HL treatment and ended at the date of diagnosis of invasive BC or ductal carcinoma in situ (DCIS), date of death, or the censoring date (on the basis of completeness of NCR data, or date of last medical information), whichever occurred first. If not otherwise stated, BC encompasses both invasive BC and DCIS. BC diagnoses within 5 years after HL treatment (n = 2) were ignored, but these survivors did contribute person-time to our analyses from 5 years after HL treatment onward, as they were still at risk to develop contralateral BC. Any subsequent cancer after HL other than BC was ignored in all analyses. Calculation of expected BC numbers was based on age- and calendar year–specific BC incidence rates in the female Dutch population (derived from the NCR), multiplied by the corresponding number of person-years at risk observed in our cohort. From the observed and expected numbers of BCs, standardized incidence ratios (SIRs), absolute excess risks (AERs, per 10,000 person-years), and their corresponding 95% CIs were computed using standard methods.^29^

Cumulative BC incidence was estimated with death as a competing risk. Cumulative BC incidences according to period of HL treatment (1975-1986, 1987-1997, and 1998-2008) were compared using competing hazards regression models.^30^

In all multivariable analyses, the cumulative doxorubicin dose was included either continuously or as a categorical variable (either as doxorubicin yes/no, or categorized as: no doxorubicin, 1-200 mg/m^2^, and >200 mg/m^2^; Data Supplement, Table S1, online only).

Cumulative procarbazine dose was categorized as ≤4.2 g/m^2^, 4.3 to 8.4 g/m^2^, or >8.4 g/m^2^. RT to the chest was categorized according to the volume of breast tissue within the radiation field as follows: no chest RT (may include neck, supraclavicular, and/or infraclavicular fields), mediastinal field (not including axilla), mantle field or RT including axillary field(s) (MF/axillary RT), and unspecified RT fields. For analysis of effect modification, gonadotoxic treatment was defined as having received pelvic RT and/or a procarbazine dose >4.2 g/m^2^. Of all survivors treated with anthracyclines, 170 (13.3%) had received epirubicin. The association between epirubicin and BC risk was evaluated using receipt of epirubicin as a binary variable (yes/no), as 89% of epirubicin-treated survivors received a cumulative dose ≥420 mg/m^2^.

To identify treatment factors associated with BC risk, we used multivariable Cox proportional hazards regression, with attained age as the time scale. The proportional hazard assumption was assessed using graphical and residual-based methods. Age at HL diagnosis violated the proportionality assumption and analyses were therefore stratified for age at HL treatment as a continuous variable.

Interactions between doxorubicin and age at HL diagnosis, gonadotoxic treatment, MF/axillary RT, and time since HL treatment were evaluated by comparing models with and without a cross-product. For assessment of effect modification by time since treatment, a cross-product between doxorubicin and various cutpoints for time since treatment (either 15, 20, 25, or 30 years after treatment) was added to the model; models were compared using the Akaike's information criterion.

We performed several sensitivity analyses: (1) excluding DCIS; (2) excluding all survivors with missing data on treatment variables (complete case analysis); (3) reclassifying survivors treated with lung RT to the MF/axillary RT category; and (4) excluding survivors treated before 1989 to see whether our results were consistent when restricted to a more recent treatment era and NCR-reported BCs.

All reported *P* values are two-sided; *P* values <.05 were considered statistically significant. All analyses were performed with Stata statistical software, version 15.0 (StataCorp, College Station, TX).

## RESULTS

The median age at first HL treatment was 27.8 years (IQR, 21.9-35.2; Table 1). Most HL survivors (69.8%) were treated with a combination of RT and chemotherapy; 11.4% received chemotherapy only. A total of 1,113 survivors received doxorubicin. Among the survivors who received chemotherapy, the proportion of survivors treated with doxorubicin increased from 32.6% between 1975 and 1986 to 84.5% between 1998 and 2008 (Data Supplement, Table S2). RT involving MF or including axillary field(s) decreased from 80.3% between 1975 and 1986 to 17.3% between 1998 and 2008. Receipt of gonadotoxic treatment decreased from 51.4% between 1975 and 1986 to 6.8% between 1998 and 2008.

**TABLE 1.** Characteristics of Female 5-Year Hodgkin Lymphoma Survivors

Survivor Characteristic	Total (N = 1,964), No. (%)	BC Event^a^ (n = 252), No. (%)
Age at first HL treatment, years		
<21	416 (21.2)	76 (30.2)
21-30	730 (37.2)	106 (42.1)
>30	818 (41.7)	70 (27.8)
Period of HL treatment		
1975-1986	539 (27.4)	101 (40.1)
1987-1997	677 (34.5)	110 (34.5)
1998-2008	748 (38.1)	41 (16.3)
Anthracycline-containing chemotherapy^b^		
No doxorubicin and no epirubicin	692 (35.2)	128 (50.8)
Doxorubicin	1,113 (56.7)	111 (44.0)
Epirubicin and no doxorubicin	159 (8.1)	13 (5.2)
Cumulative doxorubicin dose^c^		
No doxorubicin	851 (43.3)	141 (56.0)
1-200 mg/m^2^ (median 150 mg/m^2^)	448 (22.8)	38 (15.1)
201-349 mg/m^2^ (median 250 mg/m^2^)	532 (27.1)	66 (26.2)
350-700 mg/m^2^ (median 400 mg/m^2^)	89 (4.5)	5 (2.0)
Unknown dose	44 (2.2)	2 (0.8)
Chest radiotherapy^d^		
No chest RT	388 (19.8)	23 (9.1)
Mediastinal field, not including axilla	609 (31.0)	45 (17.9)
Mantle field or RT including axillary field(s)	932 (47.5)	176 (69.8)
Unspecified RT fields	35 (1.8)	8 (3.2)
Gonadotoxic treatment^e^		
No procarbazine and no pelvic RT	967 (49.2)	128 (50.8)
Procarbazine ≤4.2 g/m^2^ (cumulative dise), no pelvic RT	427 (21.7)	60 (23.8)
Procarbazine 4.3-8.4 g/m^2^ (cumulative dose), no pelvic RT	302 (15.4)	40 (15.9)
Procarbazine >8.4 g/m^2^ (cumulative dose), no pelvic RT	96 (4.9)	8 (3.2)
Pelvic RT, with/without procarbazine	112 (5.7)	10 (4.0)
No pelvic RT, unknown CT/procarbazine dose	60 (3.1)	6 (2.4)
Follow-up duration, years		
5-9	155 (7.9)	0 (0.0)
10-19	684 (34.8)	33 (13.1)
20-29	846 (43.1)	142 (56.4)
≥30	279 (14.2)	77 (30.6)
Vital status at the end of follow-up		
Alive	1,519 (77.3)	177 (70.2)
Deceased	445 (22.7)	75 (29.8)

Abbreviations: ABVD, doxorubicin, bleomycin, vinblastine, and dacarbazine; BC, breast cancer; BEACOPP, bleomycin, etoposide, doxorubicin, cyclophosphamide, vincristine, procarbazine, and prednisone; CT, chemotherapy; EBVP, epirubicin, bleomycin, vincristine, and prednisone; HL, Hodgkin lymphoma; MOPP, mechlorethamine, vincristine, procarbazine, and prednisone; MOPP-ABV, MOPP with doxorubicin, bleomycin, and vinblastine; RT, radiotherapy.

^a^ Either ductal carcinoma in situ or invasive BC, whichever occurred first.

^b^ One survivor treated without doxorubicin received mitoxantrone; one survivor received both doxorubicin and mitoxantrone; one survivor received doxorubicin and daunorubicin. Of all survivors who received epirubicin, 151 (88.9%) received six cycles of EBVP with a cumulative epirubicin dose of 420 mg/m^2^.

^c^ A doxorubicin dose of >200 mg/m^2^ corresponds to ≥6 cycles of MOPP-ABV, >4 cycles of ABVD, or ≥6 cycles of escalated BEACOPP.

^d^ The category no chest RT includes 58 survivors with RT limited to neck and 54 survivors with supraclavicular and/or infraclavicular fields (with or without neck). RT to the lung was also applied in 12 survivors in the mantle field category, in 19 survivors in the mediastinal RT category, and in one survivor in the unspecified RT category. Of all survivors categorized into unspecified RT, four survivors received supradiaphragmatic fields.

^e^ A procarbazine dose of ≤4.2 g/m^2^ corresponds to ≤3 MOPP cycles or ≤6 cycles of MOPP-ABV. A procarbazine dose of 4.3 to 8.4 g/m^2^ corresponds to 4-6 cycles of MOPP. A procarbazine dose of >8.4 g/m^2^ corresponds to >6 cycles of MOPP.

After a median follow-up of 21.6 years (IQR, 15.8-27.1 years), 252 women developed BC, 51 of whom had DCIS as a first BC event (Data Supplement, Table S3). The median interval between HL treatment and BC diagnosis was 19.9 years (IQR, 15.9-24.9 years) and the median age at BC diagnosis was 45.8 years (IQR, 40.6-51.8 years).

**FIG 1.**
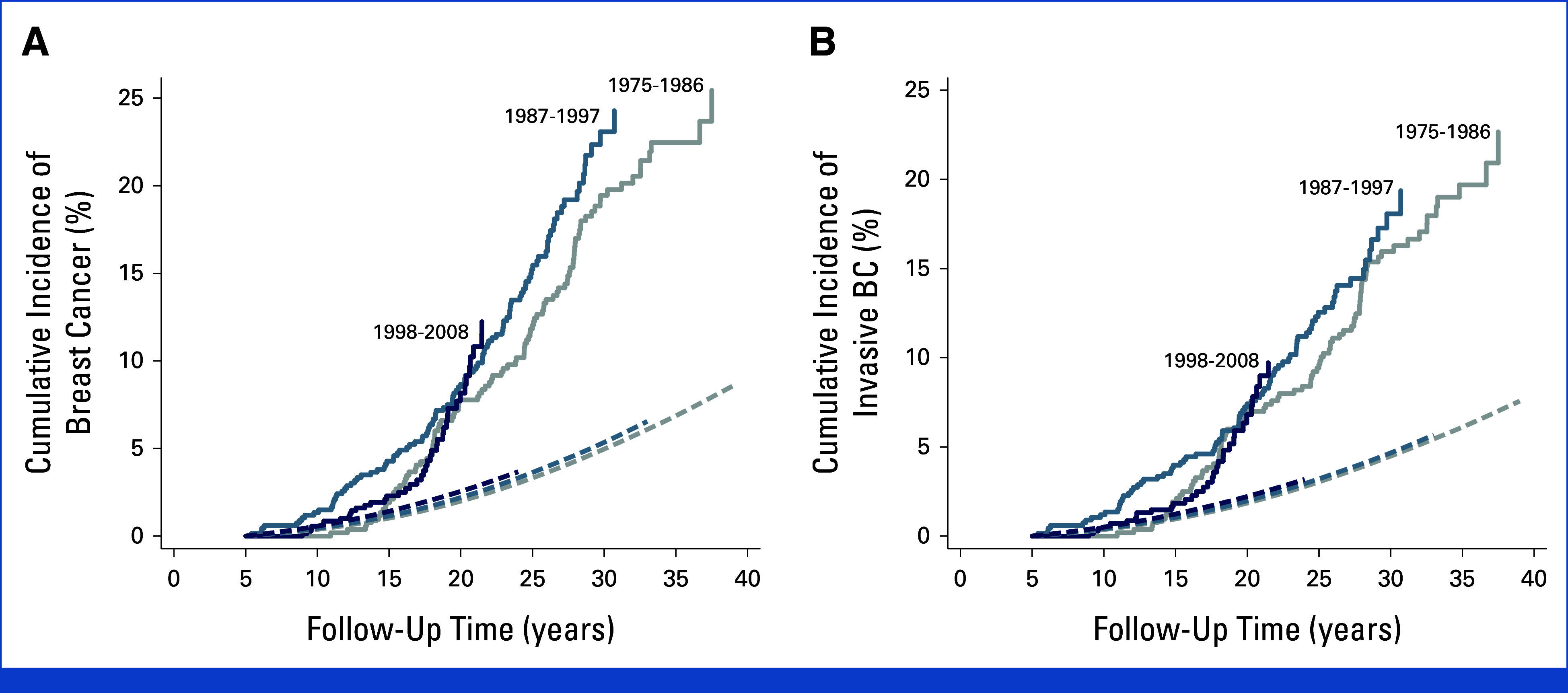
(A) Cumulative incidence of BC in female 5-year HL survivors according to HL treatment period. Solid lines represent the cumulative incidence of invasive BC and DCIS in our cohort, stratified by period of HL treatment (*P*_heterogeneity_ = .53). Dashed lines represent the expected incidence of invasive BC and DCIS in the Dutch general population, stratified by the same periods. (B) Cumulative incidence of invasive BC in female HL survivors according to HL treatment period. Solid lines represent the cumulative incidence of invasive BC in our cohort, stratified by period of HL treatment (*P*_heterogeneity_ = .65). Dashed lines represent the expected incidence of invasive BC in the general population, stratified by the same periods. BC, breast cancer; DCIS, ductal carcinoma in situ; HL, Hodgkin lymphoma.

The cumulative 30-year BC incidence was 20.8% (95% CI, 18.2 to 23.4), while a cumulative incidence of approximately 5% was expected on the basis of Dutch general population incidence rates (Fig 1). Cumulative BC incidence did not differ between treatments periods; the 20-year cumulative incidences were 7.8% (95% CI, 5.7 to 10.3), 8.7% (95% CI, 6.6 to 11.0), and 8.2% (95% CI, 5.6 to 11.3) for survivors treated between 1975 and 1986, between 1987 and 1997, and between 1998 and 2008, respectively.

Compared with the female general population, HL survivors experienced a 4.4-fold (95% CI, 3.9 to 5.0) increased BC risk, translating into an AER of 63.5 BCs per 10,000 person-years. SIRs in all treatment periods increased in the first 15 years of follow-up and then stabilized (Data Supplement, Fig S1). SIRs for BC strongly decreased with older age at diagnosis and were 17.8 (95% CI, 14.0 to 22.3), 6.7 (95% CI, 5.5 to 8.10), and 1.9 (95% CI, 1.4 to 2.4) for survivors age younger than 21 years, 21-30 years, and 31-50 years at HL treatment, respectively. Among doxorubicin-treated survivors, SIRs were significantly increased after ≥20 years of follow-up, but not in the 5-19 years of follow-up interval (adjusted for chest RT and gonadotoxic treatment, *P*_interaction_ = .001; Table 2).

**TABLE 2.** SIR and Absolute Excess Risk of BC, by HL Treatment and Attained Follow-Up

Doxorubicin	Chest RT	Gonadotoxic Treatment, Cumulative Dose	5-19 Years of Follow-Up				≥20 Years of Follow-Up			
			Total^a^	BC	SIR (95% CI)	AER (95% CI)	Total^a^	BC	SIR (95% CI)	AER (95% CI)
No doxorubicin	No chest RT	No pelvic RT and ≤4.2 g/m^2^ procarbazine	56	3	2.6 (0.5 to 7.7)	26.0 (–7.3 to 106.8)	27	1	3.1 (0.1 to 17.0)	61.7 (–27.7 to 481.2)
	No chest RT	Pelvic RT or >4.2 g/m^2^ procarbazine	51	0	—	—	38	3	2.5 (0.5 to 7.4)	52.8 (–16.2 to 219.9)
	Mediastinal RT	No pelvic RT and ≤4.2 g/m^2^ procarbazine	74	2	1.1 (0.1 to 4.0)	1.9 (–15.5 to 53.6)	39	3	4.0 (0.8 to 11.8)	104.2 (–5.7 to 370.5)
	Mediastinal RT	Pelvic RT or >4.2 g/m^2^ procarbazine	58	1	0.9 (0.0 to 5.0)	–1.8 (–15.5 to 62.8)	36	0	—	—
	MF/axillary RT	No pelvic RT and ≤4.2 g/m^2^ procarbazine	403	52	7.0 (5.2 to 9.2)	83.1 (58.5 to 113.3)	273	45	6.7 (4.9 to 8.9)	182.8 (124.6 to 255.4)
	MF/axillary RT	Pelvic RT or >4.2 g/m^2^ procarbazine	169	11	3.5 (1.7 to 6.2)	35.1 (10.4 to 74.0)	119	13	3.9 (2.1 to 6.6)	94.9 (35.1 to 185.9)
Doxorubicin	No chest RT	No pelvic RT and ≤4.2 g/m^2^ procarbazine	196	8	2.5 (1.1 to 4.8)	24.4 (1.0 to 64.4)	38	0	—	—
	No chest RT	Pelvic RT or >4.2 g/m^2^ procarbazine	71	2	1.4 (0.2 to 5.1)	7.1 (–14.0 to 70.0)	40	4	5.2 (1.4 to 13.3)	142.2 (14.0 to 417.0)
	Mediastinal RT	No pelvic RT and ≤4.2 g/m^2^ procarbazine	411	20	2.8 (1.7 to 4.3)	26.2 (10.2 to 48.6)	153	15	8.3 (4.7 to 13.7)	221.0 (110.4 to 384.0)
	Mediastinal RT	Pelvic RT or >4.2 g/m^2^ procarbazine	57	1	1.0 (0.0 to 5.7)	0.3 (–13.2 to 63.8)	35	3	5.0 (1.0 to 14.5)	119.8 (0.7 to 408.4)
	MF/axillary RT	No pelvic RT and ≤4.2 g/m^2^ procarbazine	230	17	3.8 (2.2 to 6.1)	42.9 (18.6 to 78.0)	128	17	8.2 (4.8 to 13.1)	232.5 (121.9 to 391.7)
	MF/axillary RT	Pelvic RT or >4.2 g/m^2^ procarbazine	99	6	4.5 (1.6 to 9.8)	43.3 (8.0 to 108.7)	48	13	16.0 (8.5 to 27.4)	453.1 (227.2 to 796.3)

NOTE. SIRs were calculated if at least one BC was observed.

Abbreviations: AER, absolute excess risk; BC, number of breast cancers observed; HL, Hodgkin lymphoma, MF/axillary RT, mantle field or RT including axillary field(s); RT, radiotherapy; SIR standardized incidence ratio.

^a^ Total number of observations exceed total number of survivors in the cohort, as survivors can contribute person-time to both the time interval <20 years and ≥20 years after diagnosis.

Compared with survivors who did not receive doxorubicin (43.3%), survivors treated with doxorubicin had a 1.4-fold (95% CI, 1.04 to 1.9) increased hazard of BC (adjusted for age at HL diagnosis, chest RT, and gonadotoxic treatment; Table 3, model A). The hazard ratios (HRs) for BC in survivors treated with 1-200 mg/m^2^ or >200 mg/m^2^ doxorubicin were 1.4 (95% CI, 0.9 to 2.0) and 1.5 (95% CI, 1.08 to 2.1), respectively, compared with survivors not treated with doxorubicin (Table 3, model B). Each 100 mg/m^2^ increase in doxorubicin dose was associated with a 1.18-fold increase in BC risk (95% CI, 1.05 to 1.32; Table 3, model C). In an analysis that only included survivors who had received doxorubicin (n = 1,113), the HR for BC associated with a 100 mg/m^2^ increase in doxorubicin dose (HR, 1.20 [95% CI, 0.92 to 1.56]; Data Supplement, Table S4) was very similar, albeit no longer statistically significant (*P*_trend_ = .18). Receipt of epirubicin was not associated with BC risk (HR, 0.8 [95% CI, 0.4 to 1.5]). Survivors treated with MF/axillary RT had a 2.1-fold (95% CI, 1.3 to 3.3) increased BC risk compared with survivors not treated with chest RT; BC risk was not increased in survivors treated with mediastinal RT (HR, 1.0 [95% CI, 0.6 to 1.8]). A procarbazine dose of >8.4 g/m^2^ or receipt of pelvic RT was associated with decreased BC risk (HR, 0.5 [95% CI, 0.3 to 0.9]).

**TABLE 3.** Treatment-Related Risk Factors for Subsequent BC in Female 5-Year Survivors of Hodgkin Lymphoma: Results From Multivariable Cox Regression Analysis

Treatment Variable	Total (N = 1,964), No. (%)	BC Events (n = 252), No.	Model A, HR (95% CI)	Model B, HR (95% CI)	Model C, HR (95% CI)
Doxorubicin					
No	851 (43.3)	141	1.0 (ref)		
Yes	1,113 (56.7)	111	1.4 (1.04 to 1.9)		
Doxorubicin dose, mg/m^2^^a^					
No doxorubicin	851 (43.3)	141		1.0 (ref)	
1-200	448 (22.8)	38		1.4 (0.9 to 2.0)	
201-700	621 (31.6)	71		1.5 (1.08 to 2.1)	
Unknown dose	44 (2.2)	2		—	
Doxorubicin dose (continuous)					
No doxorubicin	851 (43.3)	141			1.0 (ref)
Doxorubicin dose (per 100 mg/m^2^)	1,069 (54.4)	109			1.18 (1.05 to 1.32)
Unknown doxorubicin dose	44 (2.2)	2			—
Chest radiotherapy^b^					
No chest RT	388 (19.8)	23	1.0 (ref)	1.0 (ref)	1.0 (ref)
Mediastinum, not including axilla	609 (31.0)	45	1.0 (0.6 to 1.8)	1.0 (0.6 to 1.8)	1.1 (0.6 to 1.9)
Mantle field or RT including axillary field(s)	932 (47.5)	176	2.1 (1.3 to 3.3)	2.1 (1.3 to 3.4)	2.1 (1.3 to 3.4)
Unspecified RT fields	35 (1.8)	8	2.8 (1.2 to 6.5)	3.1 (1.3 to 7.2)	3.2 (1.3 to 7.4)
Gonadotoxic treatment^c^					
No pelvic RT and ≤4.2 g/m^2^ procarbazine	1,394 (71.0)	188	1.0 (ref)	1.0 (ref)	1.0 (ref)
No pelvic RT and 4.3-8.4 g/m^2^ procarbazine	302 (15.4)	40	0.8 (0.5 to 1.2)	0.8 (0.5 to 1.2)	0.8 (0.5 to 1.2)
Pelvic RT or >8.4 g/m^2^ procarbazine	208 (10.6)	18	0.5 (0.3 to 0.9)	0.5 (0.3 to 0.9)	0.5 (0.3 to 0.9)
No pelvic RT and unknown CT or procarbazine dose	60 (3.1)	6	0.4 (0.1 to 0.98)	0.7 (0.2 to 2.0)	0.7 (0.2 to 1.9)

NOTE. Analyses were stratified for age at first HL treatment (continuous). Lower limits of 95% CIs were rounded down, upper limits were rounded up. For doxorubicin-treated survivors, the median dose was 210 mg/m^2^ (IQR, 175-300 mg/m^2^). For doxorubicin-treated survivors with an event, the median dose was 210 mg/m^2^ (IQR, 200-280 mg/m^2^).

Abbreviations: ABVD, doxorubicin, bleomycin, vinblastine, and dacarbazine; BC, breast cancer; BEACOPP, bleomycin, etoposide, doxorubicin, cyclophosphamide, vincristine, procarbazine, and prednisone; CT, chemotherapy; MOPP, mechlorethamine, vincristine, procarbazine, and prednisone; MOPP-ABV, MOPP with doxorubicin, bleomycin, and vinblastine; RT, radiotherapy.

^a^ A doxorubicin dose of >200 mg/m^2^ corresponds to ≥6 cycles of MOPP-ABV, >4 cycles of ABVD, or ≥6 cycles of escalated BEACOPP.

^b^ The category no chest RT includes 58 survivors with RT limited to the neck and 54 survivors with supraclavicular and/or infraclavicular fields (with or without neck). RT to the lung was also applied in 12 survivors in the mantle field category, in 19 survivors in the mediastinal RT category, and in one survivor in the unspecified RT category. Of all survivors categorized into unspecified RT, four survivors received supradiaphragmatic fields.

^c^ A procarbazine dose of ≤4.2 g/m^2^ corresponds to ≤3 MOPP cycles or ≤6 cycles of MOPP-ABV. A procarbazine dose of 4.3 to 8.4 g/m^2^ corresponds to 4-6 cycles of MOPP. A procarbazine dose of >8.4 g/m^2^ corresponds to >6 cycles of MOPP.

**FIG 2.**
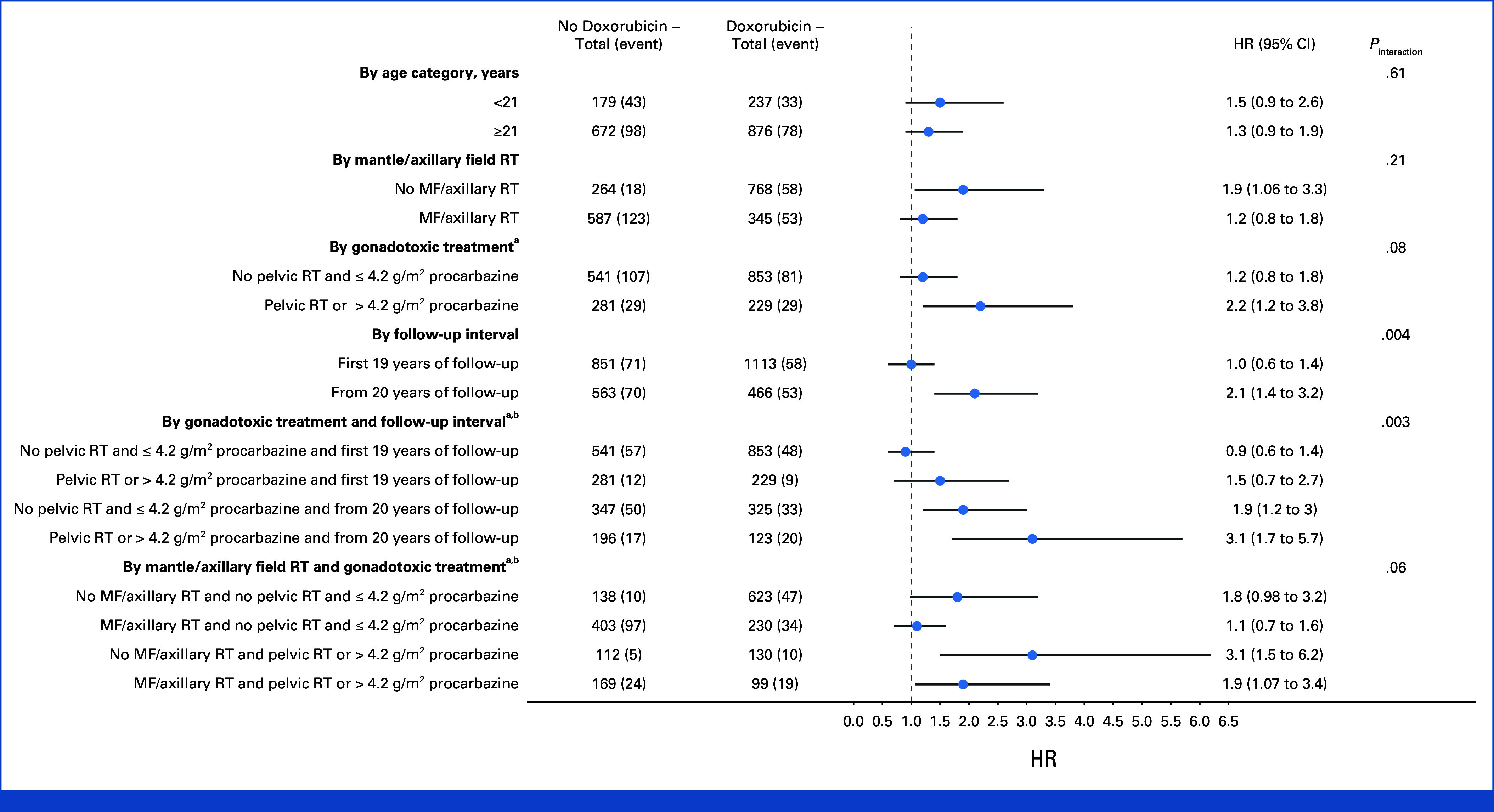
Doxorubicin and BC risk: results from multivariable Cox regression interaction analysis. Doxorubicin and BC risk, according to age at treatment, mantle or axillary field RT, gonadotoxic treatment and follow-up interval: results from multivariable Cox regression interaction analysis. All models were adjusted for chest radiotherapy, gonadotoxic treatment, and stratified for age at HL treatment. ^a^Survivors with unknown procarbazine dose are not shown (n = 60 with six events). ^b^Numbers of observations exceed total number of survivors in the cohort, as survivors can contribute person-time to both the time interval <20 years and ≥20 years after diagnosis. The model including an interaction term of doxorubicin with time since treatment, and doxorubicin and gonadotoxic treatment, was not better than a model only including interaction of doxorubicin with time since treatment (*P*_likelihood ratio test_ = .14). The model including an interaction term of doxorubicin with mantle/axillary field RT, and doxorubicin and gonadotoxic treatment, was not better than a model only including interaction of doxorubicin with mantle/axillary field RT (*P*_likelihood ratio test_ = .10). BC, breast cancer; HL, Hodgkin lymphoma; MF/axillary RT, mantle field or radiotherapy including axillary field(s).

The association between doxorubicin and BC risk differed by time since HL treatment, with increased BC risk limited to the follow-up interval ≥20 years after first HL treatment (HR_≥20 years follow-up_, 2.1 [95% CI, 1.4 to 3.2] *P*_interaction_ = .004; Fig 2). When doxorubicin was included in the model using dose categories, this effect modification by time was also present (HR_1-200 mg/m^2^__, <20 years follow-up_, 1.0 [95% CI, 0.5 to 1.6]; HR_1-200 mg/m^2^__, ≥20 years follow-up_, 2.1 [95% CI, 1.1 to 3.9]; HR_>200 mg/m^2^__, <20 years follow-up_, 1.0 [95% CI, 0.6 to 1.5]; HR_>200 mg/m^2^, ≥20 years follow-up_, 2.4 [95% CI, 1.5 to 3.7], *P*_interaction_ = .006; Data Supplement, Table S5). Gonadotoxic treatment also appeared to modify the association of doxorubicin with BC risk, although this was not statistically significant (HR_with gonadotoxic treatment_, 2.2 [95% CI, 1.2 to 3.8] HR_without gonadotoxic treatment_, 1.2 [95% CI, 0.8 to 1.8] *P*_interaction_ = .08). There was no significant effect modification by age at HL treatment; the HR_age <21 years_ was 1.5 [95% CI, 0.9 to 2.6] and HR_age ≥21 years_ 1.3 [95% CI, 0.9 to 1.9]). Receipt of MF/axillary RT did also not significantly modify the association between doxorubicin and BC risk (HR_without MF/axillary RT_ 1.9 [95% CI, 1.06 to 3.3]; HR_with MF/axillary RT_, 1.2 [95% CI, 0.8 to 1.8]).

Similar results were obtained in analyses excluding DCIS, or excluding all survivors with missing data on treatment variables, or when reclassifying survivors treated with lung RT to the MF/axillary RT category (Data Supplement, Tables S6-S8). The results of a sensitivity analysis restricted to survivors treated in the period between 1989 and 2008 were very similar to the results obtained for the full cohort (Data Supplement, Table S9).

## DISCUSSION

This large cohort study with detailed information on primary and relapse treatment, and near-complete, long-term follow-up for subsequent BCs shows that doxorubicin exposure in HL survivors treated at age 15-50 years is associated with a 1.4-fold increased BC risk. This increased risk was independent of age at first HL treatment and receipt of chest RT. In accordance with previous studies, BC risk was lower for survivors treated with radiation limited to the mediastinum compared with MF or axillary field irradiation.^1,2^ However, despite lower chest RT volumes and doses in more contemporary HL treatment regimens, neither cumulative BC incidence nor SIRs declined in more recently treated patients. This suggests that other changes in HL treatment policy counteract the effects of less toxic RT regimens.

The absence of a decline in BC incidence in more recently treated HL survivors may partly result from the decreased use of gonadotoxic treatments. As shown previously, a premature menopause, caused by gonadotoxic chemotherapy or pelvic RT, substantially decreases the risk of radiation-associated BC.^1,2,10,31^ A second major change over the past decades is the increased use of the anthracycline doxorubicin. Doxorubicin can cause malignant transformation and mutations in vitro and has been associated with an increased risk of acute myeloid leukemia in humans.^32,33^ Doxorubicin causes DNA damage by inducing double-strand breaks, and chromatin damage through histone eviction in the genome, which may explain its carcinogenicity.^34^ Animal studies support an anthracycline-BC association, with positive findings in mice and rats.^3,34-37^ An association between anthracyclines and increased BC risk has been reported in three CCS cohorts.^18-22^ The study in Dutch CCS reported that doxorubicin was associated with a dose-dependent increase in BC risk with HRs of 1.1, 2.6, and 5.8 for survivors who had received doxorubicin doses of ≤270 mg/m^2^, 271-443 mg/m^2^, and >443 mg/m^2^, respectively (P_trend_<0.001).^19^ We also found a significantly increased BC risk in survivors treated with a doxorubicin dose >200 mg/m^2^ (HR, 1.5 [95% CI, 1.08 to 2.1]) and an increasing BC risk with increasing doxorubicin dose (HR, 1.18 [95% CI, 1.05 to 1.32] per 100 mg/m^2^ increase in doxorubicin dose). When dose-response analyses were restricted to survivors treated with doxorubicin, the dose-response relationship was no longer statistically significant, but the HR point estimate for each 100 mg/m^2^ increase in doxorubicin dose did not differ from the HR on the basis of analyses including survivors not treated with doxorubicin (HR, 1.20 [95% CI, 0.92 to 1.56]). Our findings corroborate the carcinogenic potential of doxorubicin and suggest that the increased use of doxorubicin may partly explain why HL survivors treated in more recent years do not experience lower BC risk than survivors treated before the 1990s, when MF irradiation was widely used.^17^

BC surveillance for female survivors treated with MF irradiation was gradually introduced in several Dutch hospitals between 1996 and 2010, because of increased awareness of increased BC risk in these survivors.^24,38,39^ Mammography-based screening was offered to women treated with chest RT from age 25 years (≥8 years after treatment),^40^ which led to a greater proportion of DCIS diagnoses among the BCs detected in our population. A gradual increase in BC incidence, associated with increasing screening uptake in more recent years, might introduce a spurious association with doxorubicin exposure, as use of doxorubicin increased in the same time period. However, in an analysis restricted to invasive BC, results were very similar to our main analysis on the basis of all BC events. Additionally, the association between doxorubicin exposure and BC was slightly stronger among survivors not treated with chest RT. As these women were not eligible for BC screening, this argues against screening bias as an explanation for our results.

In two CCS studies, the BC risk increase and doxorubicin dose-response trend were stronger for CCS with primary tumors typically observed within the spectrum of the Li-Fraumeni (like) syndrome (LFS), such as leukemia, sarcoma, and brain tumors, compared with survivors of other solid tumors and lymphoma combined, which might indicate a gene-anthracycline interaction in the development of BC.^18,19^ Our findings show that the doxorubicin-BC association is not restricted to patients with LFS, as HL is not a LFS-associated malignancy. Additionally, the St Jude Lifetime cohort study recently showed that a higher anthracycline dose is associated with increased BC risk independent of mutations in known cancer predisposition genes.^22^ Nevertheless, further research is needed to assess whether genetic or metabolic factors may play a role in the doxorubicin-BC association.

In our study, epirubicin treatment was not associated with increased BC risk; however, only few survivors (159 females, of whom six developed BC) received anthracycline-containing regimens with epirubicin alone, many of whom were enrolled in the EORTC H7 trial that aimed to reduce toxicity without jeopardizing efficacy.^41^ The low number of survivors treated with epirubicin resulted in a low number of BCs. The upper confidence bound of our effect estimate does not preclude an increased risk associated with epirubicin exposure.

The association of doxorubicin with BC risk appeared to be present only in the interval ≥20 years after HL treatment and not in the interval 5-19 years after treatment. Increased SIRs were also limited to survivors with ≥20 years of follow-up compared with survivors with <20 years of follow-up. This may reflect an anthracycline-associated induction period for BC.

Our study included survivors from a broad treatment period and therefore takes into account the change from extended-field RT to involved-field RT. Importantly, RT regimens have significantly evolved since 2006, when the involved site/node principle was introduced and modern RT techniques, such as intensity-modulated RT or deep inspirational breath hold, were also applied to patients with HL.^42^ These therapies enable significant reductions of exposure of normal tissues to radiation^43^; therefore, a reduction in radiation-associated BC risk is expected. In balancing treatment efficacy and toxicity, studies have focused on omitting RT and trying to cure patients with early-stage HL with chemotherapy alone.^44-47^ Doxorubicin-associated BC risk may be an additional late effect that needs to be considered when weighing risks and benefits of chemotherapy only versus combined modality in primary treatment.

Our study has some limitations. Doxorubicin is commonly given in standard combinations with bleomycin and vinblastine (eg, ABV). Although there is little evidence for carcinogenicity in humans because of bleomycin or vinblastine,^32^ we could not distinguish the effect of doxorubicin from other agents. A large proportion of HL survivors had received chest RT, which made it challenging to fully separate associations between doxorubicin and BC and chest RT and BC. However, mantle or axillary field irradiation did not modify the association between doxorubicin and BC risk. In our analyses, doxorubicin and procarbazine doses were based on standard regimen doses and number of chemotherapeutic cycles; data on dose reductions were not systematically collected. However, in previous case-control studies in our cohort,^10^ dose reductions occurred infrequently. Duration of ovarian function after HL treatment would probably have been a better indicator of individual gonadotoxicity than receipt of gonadotoxic treatment; however, information on menopausal age was incomplete for a large part of the cohort.

In conclusion, our study suggests that adolescent and adult female 5-year HL survivors treated with doxorubicin have an increased risk of BC, which is independent of age at first HL treatment, receipt of chest RT, and gonadotoxic treatment. Female patients with HL receiving more contemporary treatments continue to have an increased BC risk compared with the general population, despite efforts to limit radiation-associated subsequent cancers. Our study shows that it remains of great importance to follow patients for more than 20 years after their treatment has been completed. Our findings confirm the importance of risk-based long-term follow-up care for lymphoma survivors and possibly survivors of other cancers treated with doxorubicin. Our results are also relevant for treatment strategies for patients with newly diagnosed HL, when balancing the risks and benefits of systemic therapy and RT. Since effective novel agents (eg, antibody-drug conjugates and immune checkpoint inhibitors) have been introduced in the treatment for patients with HL,^48^ future clinical trials should also aim at reducing the dose of doxorubicin. Further studies are needed to evaluate the effect of doxorubicin on BC risk in the absence of chest RT in more recently treated patient cohorts and to assess the role of genetic susceptibility in the development of doxorubicin-associated BC.

## References

[1] De Bruin ML, Sparidans J, van't Veer MB, et al: Breast cancer risk in female survivors of Hodgkin's lymphoma: Lower risk after smaller radiation volumes. J Clin Oncol 27:4239-4246, 2009 19667275 10.1200/JCO.2008.19.9174

[2] Swerdlow AJ, Cooke R, Bates A, et al: Breast cancer risk after supradiaphragmatic radiotherapy for Hodgkin's lymphoma in England and Wales: A national cohort study. J Clin Oncol 30:2745-2752, 2012 22734026 10.1200/JCO.2011.38.8835

[3] van Leeuwen FE, Ronckers CM: Anthracyclines and alkylating agents: New risk factors for breast cancer in childhood cancer survivors? J Clin Oncol 34:891-894, 2016 26834056 10.1200/JCO.2015.65.0465

[4] Kenney LB, Yasui Y, Inskip PD, et al: Breast cancer after childhood cancer: A report from the Childhood Cancer Survivor Study. Ann Intern Med 141:590-597, 2004 15492338 10.7326/0003-4819-141-8-200410190-00006

[5] Bhatia S, Yasui Y, Robison LL, et al: High risk of subsequent neoplasms continues with extended follow-up of childhood Hodgkin's disease: Report from the Late Effects Study Group. J Clin Oncol 21:4386-4394, 2003 14645429 10.1200/JCO.2003.11.059

[6] Moskowitz CS, Chou JF, Wolden SL, et al: Breast cancer after chest radiation therapy for childhood cancer. J Clin Oncol 32:2217-2223, 2014 24752044 10.1200/JCO.2013.54.4601 PMC4100937

[7] Travis LB, Hill D, Dores GM, et al: Cumulative absolute breast cancer risk for young women treated for Hodgkin lymphoma. J Natl Cancer Inst 97:1428-1437, 2005 16204692 10.1093/jnci/dji290

[8] Inskip PD, Robison LL, Stovall M, et al: Radiation dose and breast cancer risk in the Childhood Cancer Survivor Study. J Clin Oncol 27:3901-3907, 2009 19620485 10.1200/JCO.2008.20.7738 PMC2734395

[9] De Bruin ML, Huisbrink J, Hauptmann M, et al: Treatment-related risk factors for premature menopause following Hodgkin lymphoma. Blood 111:101-108, 2008 17890454 10.1182/blood-2007-05-090225

[10] Krul IM, Opstal-van Winden AWJ, Aleman BMP, et al: Breast cancer risk after radiation therapy for Hodgkin lymphoma: Influence of gonadal hormone exposure. Int J Radiat Oncol Biol Phys 99:843-853, 2017 28888722 10.1016/j.ijrobp.2017.07.016

[11] Canellos GP, Anderson JR, Propert KJ, et al: Chemotherapy of advanced Hodgkin's disease with MOPP, ABVD, or MOPP alternating with ABVD. N Engl J Med 327:1478-1484, 1992 1383821 10.1056/NEJM199211193272102

[12] Bonadonna G, Zucali R, Monfardini S, et al: Combination chemotherapy of Hodgkin's disease with adriamycin, bleomycin, vinblastine, and imidazole carboxamide versus MOPP. Cancer 36:252-259, 1975 54209 10.1002/1097-0142(197507)36:1<252::aid-cncr2820360128>3.0.co;2-7

[13] Raemaekers JM, van der Maazen RW: Hodgkin's lymphoma: News from an old disease. Neth J Med 66:457-466, 2008 19075311

[14] Engert A, Franklin J, Eich HT, et al: Two cycles of doxorubicin, bleomycin, vinblastine, and dacarbazine plus extended-field radiotherapy is superior to radiotherapy alone in early favorable Hodgkin's lymphoma: Final results of the GHSG HD7 trial. J Clin Oncol 25:3495-3502, 2007 17606976 10.1200/JCO.2006.07.0482

[15] Engert A, Schiller P, Josting A, et al: Involved-field radiotherapy is equally effective and less toxic compared with extended-field radiotherapy after four cycles of chemotherapy in patients with early-stage unfavorable Hodgkin's lymphoma: Results of the HD8 trial of the German Hodgkin's Lymphoma Study Group. J Clin Oncol 21:3601-3608, 2003 12913100 10.1200/JCO.2003.03.023

[16] Fermé C, Eghbali H, Meerwaldt JH, et al: Chemotherapy plus involved-field radiation in early-stage Hodgkin's disease. N Engl J Med 357:1916-1927, 2007 17989384 10.1056/NEJMoa064601

[17] Schaapveld M, Aleman BM, van Eggermond AM, et al: Second cancer risk up to 40 years after treatment for Hodgkin's lymphoma. N Engl J Med 373:2499-2511, 2015 26699166 10.1056/NEJMoa1505949

[18] Henderson TO, Moskowitz CS, Chou JF, et al: Breast cancer risk in childhood cancer survivors without a history of chest radiotherapy: A report from the Childhood Cancer Survivor Study. J Clin Oncol 34:910-918, 2016 26700127 10.1200/JCO.2015.62.3314 PMC4871997

[19] Teepen JC, van Leeuwen FE, Tissing WJ, et al: Long-term risk of subsequent malignant neoplasms after treatment of childhood cancer in the DCOG LATER study cohort: Role of chemotherapy. J Clin Oncol 35:2288-2298, 2017 28530852 10.1200/JCO.2016.71.6902

[20] Veiga LH, Curtis RE, Morton LM, et al: Association of breast cancer risk after childhood cancer with radiation dose to the breast and anthracycline use: A report from the Childhood Cancer Survivor Study. JAMA Pediatr 173:1171-1179, 2019 31657853 10.1001/jamapediatrics.2019.3807 PMC6820042

[21] Turcotte LM, Liu Q, Yasui Y, et al: Chemotherapy and risk of subsequent malignant neoplasms in the Childhood Cancer Survivor Study cohort. J Clin Oncol 37:3310-3319, 2019 31622130 10.1200/JCO.19.00129 PMC7001784

[22] Ehrhardt MJ, Howell CR, Hale K, et al: Subsequent breast cancer in female childhood cancer survivors in the St Jude Lifetime Cohort Study (SJLIFE). J Clin Oncol 37:1647-1656, 2019 31075046 10.1200/JCO.18.01099 PMC6804891

[23] van Leeuwen FE, Klokman WJ, Hagenbeek A, et al: Second cancer risk following Hodgkin's disease: A 20-year follow-up study. J Clin Oncol 12:312-325, 1994 8113838 10.1200/JCO.1994.12.2.312

[24] van Leeuwen FE, Klokman WJ, Veer MB, et al: Long-term risk of second malignancy in survivors of Hodgkin's disease treated during adolescence or young adulthood. J Clin Oncol 18:487-497, 2000 10653864 10.1200/JCO.2000.18.3.487

[25] Aleman BM, van den Belt-Dusebout AW, Klokman WJ, et al: Long-term cause-specific mortality of patients treated for Hodgkin's disease. J Clin Oncol 21:3431-3439, 2003 12885835 10.1200/JCO.2003.07.131

[26] De Bruin ML, Burgers JA, Baas P, et al: Malignant mesothelioma after radiation treatment for Hodgkin lymphoma. Blood 113:3679-3681, 2009 19234144 10.1182/blood-2008-10-184705

[27] Schouten LJ, Höppener P, van den Brandt PA, et al: Completeness of cancer registration in Limburg, The Netherlands. Int J Epidemiol 22:369-376, 1993 8359950 10.1093/ije/22.3.369

[28] van der Willik KD, Ruiter R, van Rooij FJA, et al: Ascertainment of cancer in longitudinal research: The concordance between the Rotterdam Study and The Netherlands Cancer Registry. Int J Cancer 147:633-640, 2020 31642518 10.1002/ijc.32750 PMC7317466

[29] Breslow NE, Day NE: Statistical methods in cancer research. Volume II—The design and analysis of cohort studies. IARC Sci Publ 82:1-406, 1987 3329634

[30] Fine JP, Gray RJ: A proportional hazards model for the subdistribution of a competing risk. J Am Stat Assoc 94:496-509, 1999

[31] Collaborative Group on Hormonal Factors in Breast Cancer: Menarche, menopause, and breast cancer risk: Individual participant meta-analysis, including 118 964 women with breast cancer from 117 epidemiological studies. Lancet Oncol 13:1141-1151, 2012 23084519 10.1016/S1470-2045(12)70425-4 PMC3488186

[32] Overall evaluations of carcinogenicity: An updating of IARC Monographs volumes 1 to 42. IARC Monogr Eval Carcinog Risks Hum Suppl 7:1-440, 1987 3482203

[33] Marquardt H, Philips FS, Sternberg SS: Tumorigenicity in vivo and induction of malignant transformation and mutagenesis in cell cultures by adriamycin and daunomycin. Cancer Res 36:2065-2069, 1976 1268859

[34] Qiao X, van der Zanden SY, Wander DPA, et al: Uncoupling DNA damage from chromatin damage to detoxify doxorubicin. Proc Natl Acad Sci USA 117:15182-15192, 2020 32554494 10.1073/pnas.1922072117 PMC7334570

[35] Price PJ, Suk WA, Skeen PC, et al: Transforming potential of the anticancer drug adriamycin. Science 187:1200-1201, 1975 1167703 10.1126/science.1167703

[36] Solcia E, Ballerini L, Bellini O, et al: Mammary tumors induced in rats by adriamycin and daunomycin. Cancer Res 38:1444-1446, 1978 639071

[37] Bucclarelli E: Mammary tumor induction in male and female Sprague-Dawley rats by adriamycin and daunomycin. J Natl Cancer Inst 66:81-84, 1981 6935469

[38] Swerdlow AJ, Barber JA, Hudson GV, et al: Risk of second malignancy after Hodgkin's disease in a collaborative British cohort: The relation to age at treatment. J Clin Oncol 18:498-509, 2000 10653865 10.1200/JCO.2000.18.3.498

[39] Ng AK, Bernardo MV, Weller E, et al: Second malignancy after Hodgkin disease treated with radiation therapy with or without chemotherapy: Long-term risks and risk factors. Blood 100:1989-1996, 2002 12200357 10.1182/blood-2002-02-0634

[40] Nijdam A, Dekker N, Aleman BMP, et al: Setting up a national infrastructure for survivorship care after treatment for Hodgkin lymphoma. Br J Haematol 186:e103-e108, 2019 31090916 10.1111/bjh.15936

[41] Noordijk EM, Carde P, Dupouy N, et al: Combined-modality therapy for clinical stage I or II Hodgkin's lymphoma: Long-term results of the European Organisation for Research and Treatment of Cancer H7 randomized controlled trials. J Clin Oncol 24:3128-3135, 2006 16754934 10.1200/JCO.2005.05.2746

[42] Specht L, Yahalom J, Illidge T, et al: Modern radiation therapy for Hodgkin lymphoma: Field and dose guidelines from the international lymphoma radiation oncology group (ILROG). Int J Radiat Oncol Biol Phys 89:854-862, 2014 23790512 10.1016/j.ijrobp.2013.05.005

[43] Milgrom SA, Bakst RL, Campbell BA: Clinical outcomes confirm conjecture: Modern radiation therapy reduces the risk of late toxicity in survivors of Hodgkin lymphoma. Int J Radiat Oncol Biol Phys 111:841-850, 2021 34655558 10.1016/j.ijrobp.2021.06.030

[44] André MPE, Girinsky T, Federico M, et al: Early positron emission tomography response–adapted treatment in stage I and II Hodgkin lymphoma: Final results of the randomized EORTC/LYSA/FIL H10 trial. J Clin Oncol 35:1786-1794, 2017 28291393 10.1200/JCO.2016.68.6394

[45] Franklin J, Eichenauer DA, Becker I, et al: Optimisation of chemotherapy and radiotherapy for untreated Hodgkin lymphoma patients with respect to second malignant neoplasms, overall and progression-free survival: Individual participant data analysis. Cochrane Database Syst Rev 9:Cd008814, 2017 28901021 10.1002/14651858.CD008814.pub2 PMC6483617

[46] Meyer RM, Gospodarowicz MK, Connors JM, et al: ABVD alone versus radiation-based therapy in limited-stage Hodgkin's lymphoma. N Engl J Med 366:399-408, 2012 22149921 10.1056/NEJMoa1111961 PMC3932020

[47] Radford J, Barrington S, Counsell N, et al: Involved field radiotherapy versus No further treatment in patients with clinical stages IA and IIA Hodgkin lymphoma and a 'negative' PET scan after 3 cycles ABVD. Results of the UK NCRI RAPID trial. Blood 120:547, 2012

[48] Vassilakopoulos TP, Liaskas A, Pereyra P, et al: Incorporating monoclonal antibodies into the first-line treatment of classical Hodgkin lymphoma. Int J Mol Sci 24:13187, 2023 37685994 10.3390/ijms241713187 PMC10487754

